# Recent Progress of Liquid Metal-Based Electromagnetic Shielding Materials

**DOI:** 10.3390/nano15171346

**Published:** 2025-09-01

**Authors:** Jialu Suo, Li Guan, Peng Chen, Yujie Zhu, Mengmeng Lin, Yuanhua Hu, Zhen Liu, Shijie Han, Shixuan Han, Zhongyi Bai, Xiaoqin Guo, Biao Zhao, Rui Zhang

**Affiliations:** 1 Henan Key Laboratory of Aeronautical Materials and Application Technology, Henan International Joint Laboratory of Aeronautical Function Materials and Advanced Processing Technology, School of Material Science and Engineering, Zhengzhou University of Aeronautics, Zhengzhou 450046, China; 18737972661@163.com (J.S.); chen678000@163.com (P.C.); 15839700691@163.com (Y.H.); hsx0206hsx@163.com (S.H.); zhy_bai@163.com (Z.B.); zhangray@zua.edu.cn (R.Z.); 2 Institute of Advanced Ceramics, Henan Academy of Sciences, Zhengzhou 450046, China; 3 School of Microelectronics, Fudan University, Shanghai 200433, China

**Keywords:** gallium-based liquid metal, electromagnetic interference shielding, interfacial polarization

## Abstract

Electromagnetic shielding materials are pivotal for suppressing electromagnetic radiation and mitigating potential health risks that electronic devices may pose to humans. Beyond health protection, they also hold significant strategic value in safeguarding national information security and maintaining stability. In the research of electromagnetic shielding materials, continuous technological advancements and growing application demands have driven the emergence of various novel materials. Among these, liquid metal (LM) exhibits outstanding properties—including exceptional electrical conductivity, excellent fluidity, and superior deformability—which endow it with substantial potential for application in electromagnetic shielding. Looking ahead, with the continuous advancement in related technologies, liquid metal-based electromagnetic shielding materials are expected to provide effective solutions to key challenges such as electromagnetic pollution and interference. This contribution synthesizes the latest literature. First, it clarifies the nomenclature and classification of liquid metals, as well as the fundamental framework for electromagnetic shielding. Then, it systematically distills recent research advances based on four key design motifs. These motifs include monolithic liquid metal (LM) scaffolds, LM/conductive-filler blends, LM/magnetic particle composites, and architectured multifunctional architectures. Finally, this review identifies current bottlenecks in the field and outlines directions for future development, which aim to achieve ultra-lightweight, broadband, and intelligent LM-based electromagnetic shields.

## 1. Introduction

**Figure 1:**
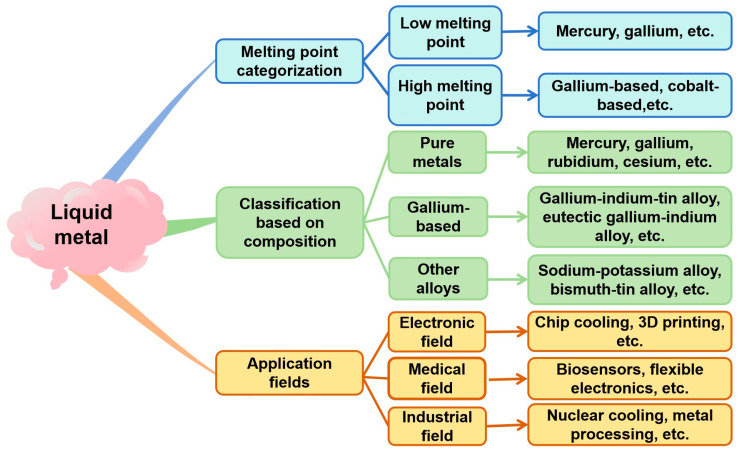
Classification of liquid metals based on composition and melting point, and application fields of liquid metals.

Liquid metals are substances that exist in a liquid state at or slightly above room temperature and possess metallic properties. In a broad sense, liquid metals refer to metals or metallic alloys that exist in a liquid form within a certain temperature range [1,2,3]. Generally speaking, liquid metals exhibit elevated boiling temperatures. For instance, the boiling point of mercury is approximately 356.73 °C [4], the boiling point of gallium is as high as 2204 °C [5]; these are significantly higher than the boiling point of water. Beyond their elevated boiling points, liquid metals distinguish themselves through excellent electrical and thermal conductivities, pronounced fluidity, and minimal surface tension. Liquid metal, due to its unique physical and chemical properties, can be simply classified according to the melting point and internal composition of the material. It can also be categorized based on differences in composition, melting point, material properties, and application fields, as shown in Figure 1.

The following is a classification of liquid metals based on their different compositions:

### 1.1. Pure Metal Liquid Metals

**Figure 2:**
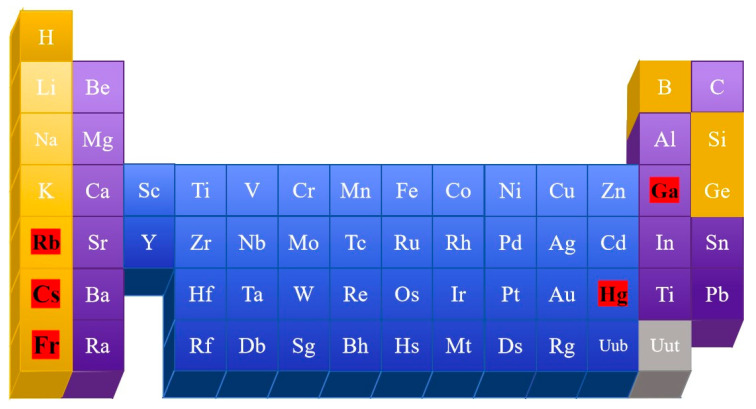
Schematic representation of elemental liquid metal.

Pure metal liquid metals refer to substances composed of a single metal element that are in a liquid state at room temperature or slightly above room temperature. They possess typical characteristics, which include pronounced electrical and thermal conductivities along with metallic luster [6,7]. Common liquid metals include Hg [8], Rb [9], Cs [10], Fr [11], and Ga [12], as illustrated in Figure 2. Among these, Hg remains the most ubiquitous liquid metal encountered in everyday settings. However, the highly toxic and volatile nature of Hg limits its development [13]; Rb undergoes an explosive reaction when in contact with air [14]. Fr and Cs are radioactive [15,16]. Therefore, these materials are not suitable for practical daily work and life.

At ambient temperature and pressure (25 °C, 1 atmosphere), mercury (Hg) is the only metallic element that exists in a liquid state [17]. In terms of physical properties, mercury (Hg) has a melting point of −38.83 °C and a boiling point of 356.73 °C. Under natural conditions, it is a silvery-white liquid with a metallic luster. It possesses excellent electrical and thermal conductivity, with an electrical conductivity near 10.6 × 10^6^ S/m and a thermal conductivity of 8.3 W/(m·K) [18]. In terms of chemical properties, mercury shows limited reactivity toward both O_2_ and H_2_O under ambient conditions. However, it rapidly reacts with sulfur to form mercuric sulfide (HgS) [19,20,21]. When the human body is exposed to mercury vapor or its compounds, it can readily cause damage to the nervous system and cellular metabolism [22].

### 1.2. Gallium-Based Liquid Metals

**Figure 3:**
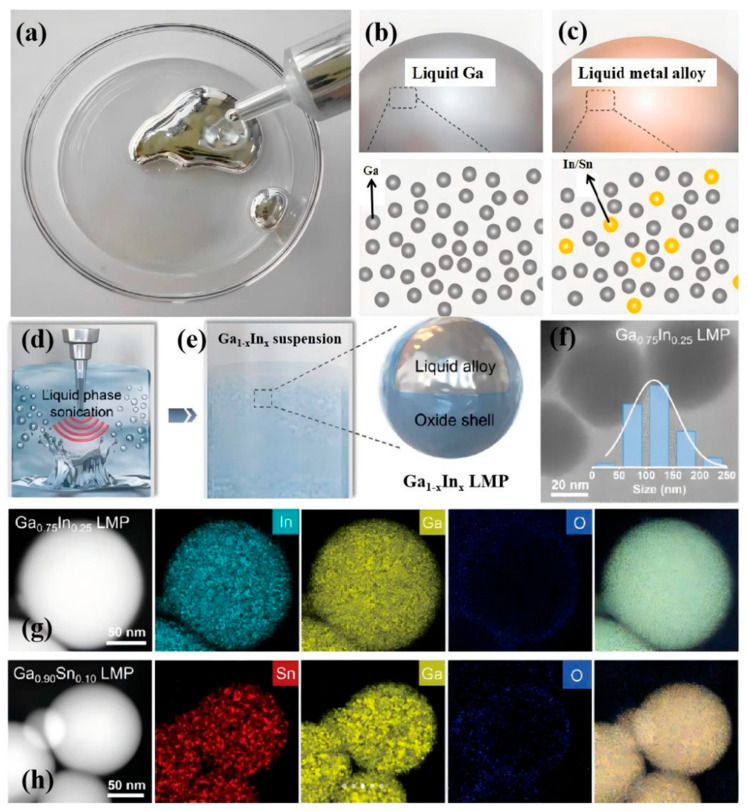
Schematic illustration of composition-tunable Ga_1−x_In_x_ and Ga_1−x_Sn_x_ binary liquid metal alloys: (**a**) Schematic of gallium-based liquid metal; (**b**,**c**) Schematic illustrating the gallium-mediated low-melting-point alloying process; (**d**,**e**) Schematic depicting ultrasonic preparation of Ga_1−x_Sn_x_ low-melting-point alloy and its core-shell structure; (**f**) Transmission electron microscopy image of Ga_0.75_Sn_0.25_ low-melting-point alloy with an inset showing the corresponding particle size distribution; (**g**,**h**) Scanning transmission electron microscopy images and corresponding energy-dispersive X-ray spectroscopy elemental mapping of Ga_0.75_Sn_0.25_ and Ga_0.90_Sn_0.10_ low-melting-point alloys. Reprinted/adapted with permission from Ref. [39]. Copyright 2024, Huang, H.; Ding, X.; Mao, X.; Yan, Y.; Lv, F.; Pan, B.; Huang, W.; Wang, L.; Han, N.; Li, Y. More details about “Copyright and Licensing” are available via the following link: https://doi.org/10.1002/adfm.202408966.

Gallium-based liquid metals refer to a type of liquid metal material primarily composed of gallium, which possesses numerous unique strengths in the area of liquid metals, as illustrated in Figure 3. From the atomic distribution and composition of pure gallium liquid metal (Figure 3b) and liquid metal alloys (Figure 3c), as well as the composition-adjustable Ga_1−x_In_x_ and Ga_1−x_Sn_x_ binary liquid metal alloys, gallium-based liquid metals exhibit a low melting point, enabling them to remain in a liquid state and maintain good fluidity at room temperature or slightly above. This characteristic allows for easy flow within containers or pipelines during processing operations, facilitating shaping and molding [14,23,24,25,26]. Moreover, gallium-based liquid metals possess a high boiling point and a broad liquidus temperature range, enabling them to withstand significant temperature variations without undergoing a phase change [27,28]. Moreover, gallium-based liquid metals retain outstanding electrical and thermal conductivities [29,30,31,32,33]. At room temperature and normal pressure, gallium quickly develops a compact oxide film when exposed to air [3,34,35]; this oxide layer can protect the underlying metal from further oxidation, thereby maintaining stability in relatively harsh environments and preventing it from readily undergoing chemical reactions with other substances [36,37]. Among their additional characteristics, gallium-based liquid metals display minimal viscosity and modest surface tension. When in contact with other materials, they encounter minimal flow resistance and can achieve better surface conformability [37,38].

### 1.3. Other Alloy-Based Liquid Metals

In addition to elemental liquid metals and gallium-containing liquid alloys, other liquid metals are most commonly found in the form of alloys, such as Na-K alloy [40,41] and Bi-Sn alloy, etc. [42,43,44,45]. These alloys have distinguished themselves through their unique properties and broad applications, emerging as typical representatives among alloy-based liquid metals. The following sections will provide a detailed introduction to these two exemplary alloy-based liquid metals.

Na-K alloy is a typical low-melting-point, highly reactive liquid metal [34]. Under ambient temperature and pressure conditions, the sodium–potassium (Na-K) alloy exhibits a silvery-white appearance. Under specific conditions, the melting point of the Na-K alloy can reach as low as −12 °C, which is significantly lower than room temperature. Additionally, it possesses excellent thermal conductivity, with a thermal conductivity as high as 85 W/(m·K). In practical applications, the Na-K alloy is used as a coolant, capable of efficiently transferring and dissipating large amounts of heat, thereby achieving highly effective heat dissipation [3,35]. In addition to its physical properties, Na-K alloy is highly chemically reactive [46]. Consequently, Na-K alloy occupies an important position among alloy-based liquid metals. Bi-Sn alloy is a typical liquid metal that is easy to process and environmentally friendly [47,48]. Under specific conditions, the melting point of Bi-Sn alloy is approximately 138 °C, which is relatively low compared to many other metallic elements and allows for rapid solidification. This characteristic endows it with a significant role in low-temperature soldering applications [49,50,51], such as the processing and soldering of electronic components [44]. The electrical conductivity of Bi-Sn alloy is approximately 1 × 10^6^ S/m, and its thermal conductivity is around 30 W/(m·K). Although these values are lower than those of pure metals, the alloy exhibits stable electrical and thermal performance, which can effectively meet the requirements for the production and application of multiple components [52,53,54].

In summary, Na-K alloy and Bi-Sn alloy, as typical representatives of alloy-based liquid metals, each play significant roles in different fields due to their unique physical and chemical properties [28,55,56,57].

## 2. Preparation Methods of Liquid Metal-Based Materials

The primary methods for preparing liquid metal-based materials include direct forming, composite material preparation, 3D printing technology, mechanical stirring, microfluidic control technology, vapor deposition, and ultrasonic electroplating replacement. The following is a brief overview:

### 2.1. Direct Molding Method

Among all fabrication routes for liquid metal-derived materials, direct molding stands out as the most elementary and unembellished strategy [58]. The behavior arises from the distinctive attributes of liquid metals, including their low melting points and high fluidity; this method enables the processing and shaping of materials through a relatively simple fabrication process. Specifically, under external forces such as gravity and pressure, the inherent fluidity of liquid metals is utilized to fill predefined molds. Subsequent solidification is achieved through natural cooling. The primary advantages of the direct molding method are its low cost and ease of operation, which facilitate the efficient transformation of raw materials into finished products, thereby enhancing production efficiency [59,60,61,62,63,64].

### 2.2. Composite Preparation Method

The composite preparation method is an important fabrication technique that combines liquid metals with other materials to fully exploit the advantages of each component, thereby endowing the materials with new properties or enhancing the original properties of the materials. The essence of creating the composite relies on exploiting the pronounced flowability and excellent electrical conduction inherent to liquid metals, among other characteristics, to prepare composite materials with other functional materials through physical or chemical methods [65,66,67]; depending on the type of added material, composite materials can be simply classified into several main categories, including “liquid metal–metal composites”, “liquid metal–ceramic composites”, and “liquid metal–polymer composites”. The preparation process of the composite method typically involves several key steps, including material pretreatment, mixing and dispersion, and shaping and curing. Through the composite preparation method, it is possible to achieve comprehensive properties that cannot be realized by a single material [68,69,70].

### 2.3. 3D Printing Technology

3D printing technology has brought revolutionary breakthroughs to the preparation of liquid metal-based materials, enabling the precise fabrication of complex structures and personalized customization [71,72,73]. This technology allows the loading of liquid metal and other material mixtures into specially designed printing nozzles. Through computer-controlled nozzle movement trajectories, the materials are deposited layer by layer to form the desired structures [74]. 3D printing technology overcomes the limitations of traditional manufacturing methods in producing complex structures and enables the fabrication of intricate internal architectures, such as porous and lattice structures, that are difficult to achieve with conventional techniques. This capability significantly enhances the performance of the materials.

### 2.4. Other Preparation Methods

In addition to the aforementioned preparation methods, liquid metal-based materials can also be fabricated using several specialized techniques, such as mechanical agitation, microfluidic manipulation technology, vapor deposition, and ultrasonic electroplating replacement.

Mechanical agitation employs externally applied forces, such as magnetic stirring, ultrasonic vibration, or high-shear mixers to physically disperse liquid metals. By delivering regulated mechanical energy, the native oxide skin enveloping the melt is disrupted, fragmenting the bulk into microdroplets or thin lamellae and thereby ensuring compositional homogenization [75]. Microfluidic steering employs carefully etched microchannel geometries on chip-scale devices to govern the motion, blending, and chemical transformation of liquid metals with micrometer-level accuracy. Within these networks, droplets of metallic fluid can be created, coalesced, or divided at will, and distinct reagents can be intermixed under controlled conditions. Such an approach is ideally suited for fabricating micro- and nano-scale liquid metal composites whose dimensions are tightly defined and whose chemical makeup remains homogeneous throughout [76]. Vapor deposition involves transporting gaseous metals or metal compounds to a substrate surface, where they are deposited to form liquid metal-based films or coatings through physical or chemical processes [77]. LM ultrasonic electroplating replacement involves immersing the substrate in a molten metal bath. Physical and chemical reactions occur between the substrate and the liquid metal at the interface. These reactions further induce metallurgical bonding. The immersion time and temperature curve can be controlled during the process. This control destabilizes the intrinsic oxide layer on the surface of the liquid metal, promoting uniform wetting of the substrate by the liquid metal. Uniformity of composition is achieved at the substrate-coating interface [78].

As shown in Table 1: Preparation method of liquid metal-based materials.

## 3. Electromagnetic Shielding Principles and Methods

### 3.1. The Basic Principles of Electromagnetic Shielding

**Figure 4:**
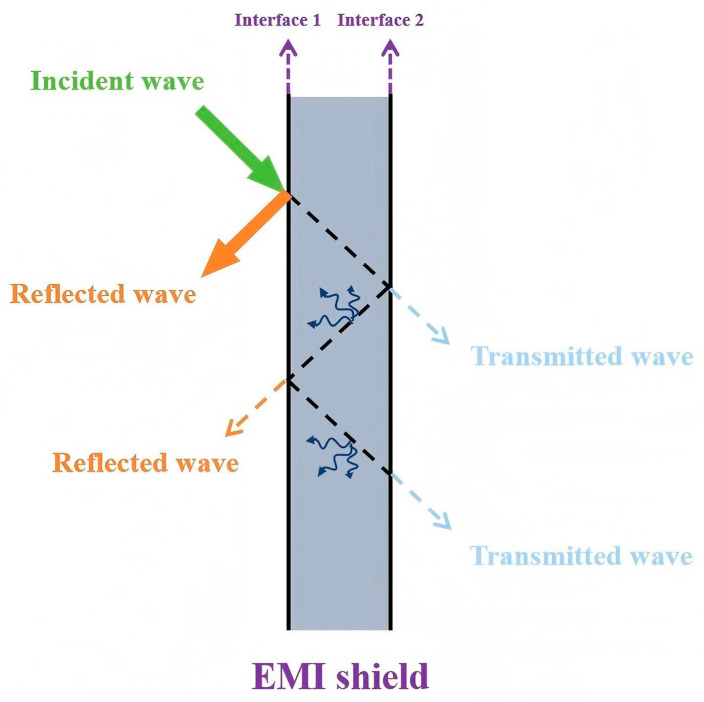
Schematic illustration of electromagnetic interference-shielding behavior.

When electromagnetic waves come into contact with a shielding layer, a portion of the waves is reflected at the surface of the shielding layer due to the impedance mismatch between the shielding layer and air. The electromagnetic waves that are not reflected will penetrate the shielding layer. During propagation within the shielding layer, the energy of the electromagnetic waves is dissipated in various forms, such as being converted into thermal energy or other forms of energy. Only a small amount of energy will pass through the shielding layer in the form of transmitted waves [79,80,81,82,83,84,85]. As shown in Figure 4.

The shielding layer’s ability to protect against incident electromagnetic waves is defined as the Electromagnetic Interference Shielding Effectiveness (EMI SE). The higher the shielding effectiveness, the better the material’s protective effect against electromagnetic waves, as shown in Equation (1) [86]:

(1) $$SE_{T}\left(\mathrm{dB}\right)=10\log_{10}\frac{P_{T}}{P_{I}}=20\log_{10}\frac{E_{T}}{E_{I}}=20\log_{10}\frac{H_{T}}{H_{I}}$$

In the formula, *P_T_* represents the intensity of the transmitted electromagnetic wave power, and *P_I_* represents the intensity of the incident electromagnetic wave power. Similarly, *E_T_* and *E_I_* denote the electric field strengths of the transmitted and incident electromagnetic waves, respectively, while *H_T_* and *H_I_* denote the magnetic field strengths of the transmitted and incident electromagnetic waves, respectively.

The total Electromagnetic Interference Shielding Effectiveness (EMI *SE_T_*) is the sum of the attenuation achieved through reflection (*SE_R_*), absorption (*SE_A_*), and multiple reflections (*SE_M_*), as shown in Equation (2) [87]:

(2) $$SE_{T}=SE_{R}+SE_{A}+SE_{M}$$

1. Reflection Loss: Reflection is one of the principal mechanisms of electromagnetic interference (EMI) shielding. Upon encountering a shielding layer, an electromagnetic wave undergoes partial reflection at the interface due to the impedance mismatch between the shielding material and the surrounding medium. The extent of reflection loss is contingent upon the material properties of the shielding layer, its thickness, and the frequency of the incident electromagnetic wave, as depicted in Equation (3) [88]:

(3) $$SE_{R}(\mathrm{dB})=39.5+10\log_{10}\frac{\sigma }{2\pi f\mu }$$

In the above equation, *σ* and *μ* represent the electrical conductivity and magnetic permeability of the shielding layer, respectively, while *f* denotes the frequency of the incident electromagnetic wave. As the electrical conductivity (*σ*) increases, the reflection loss (*SE_R_*) also increases.

2. Absorption Loss: After entering the shielding layer, the electromagnetic wave propagates within the material, where it is absorbed, leading to energy attenuation. The absorption loss primarily depends on the magnetic permeability, electrical conductivity, and thickness of the shielding material, as shown in Equation (4) [89]:

(4) $$SE_{A}(\mathrm{dB})=8.7d\sqrt{\pi f\mu \sigma }$$

In the above equation, *d*, *σ*, and *μ* represent the thickness, electrical conductivity, and magnetic permeability of the shielding layer, respectively, while *f* is the frequency of the incident electromagnetic wave. These three factors are the main determinants of absorption loss.

3. Multiple Reflection Loss: Electromagnetic waves undergo multiple reflections within the interfaces of the shielding layer. Each reflection results in a portion of the energy being absorbed or reflected back into the original medium, thereby further increasing the propagation path and loss of the electromagnetic waves, enhancing the shielding effect. This process can be repeated until the energy of the electromagnetic waves is completely dissipated, as shown in Equation (5) [90]:

(5) $$SE_{M}(\mathrm{dB})=20\log_{10}(1-e^{-\frac{2d}{\delta }})$$

In the above equation, *d* represents the thickness of the shielding layer, and *δ* denotes the skin depth.

### 3.2. Methods for Electromagnetic Shielding Testing

**Figure 5:**
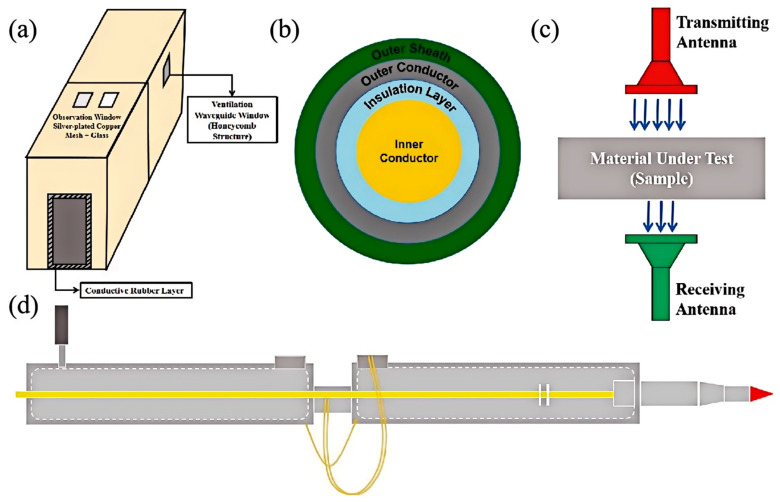
(**a**) Schematic diagram of the shielded room structure; (**b**) schematic diagram of the coaxial transmission line structure; (**c**) schematic diagram of the free-space method; (**d**) schematic diagram of the near-field probe.

In the modern technological field where the electromagnetic environment is increasingly complex, accurately assessing the electromagnetic shielding performance of materials and devices has become a crucial aspect in ensuring the stable operation of electronic systems, information security, and electromagnetic compatibility. Based on the testing principles and application scenarios, the methods for electromagnetic shielding testing are primarily divided into the shielded room method [91,92], the coaxial transmission line method [93,94,95], the free-space method [96], and the near-field probe method [3], among others. As shown in Figure 5. A detailed analysis of these methods is provided as follows:

1. Shielded Room Method: By constructing an enclosed shielding space to simulate real-world complex electromagnetic environments, this method allows for a systematic evaluation of the shielding effectiveness of large-scale equipment or entire buildings. The material to be tested is placed inside the shielded room, where electromagnetic waves of known intensity are emitted. The intensity of the electromagnetic waves that penetrate through the shielding room is measured outside. This measurement is used to assess the shielding effectiveness of the room, thereby inferring the electromagnetic shielding performance of the tested device or material.
2. Coaxial Transmission Line Method: Based on the transmission characteristics of electromagnetic waves in coaxial structures, this method utilizes coaxial transmission lines to carry electromagnetic waves. The shielding material to be tested is either fabricated into a coaxial structure or placed around a coaxial line. By measuring the power changes of the electromagnetic waves in the transmission line before and after passing through the shielding material, the shielding effectiveness can be calculated. This method focuses on the precise measurement of shielding performance for sheet-like and strip-like materials.
3. Free-Space Method: In free space, electromagnetic waves are transmitted towards the material under test, and the waves that have passed through the shielding material are received at a certain distance. By comparing the intensities of the transmitted and received signals, the shielding effectiveness is determined. This method is suitable for testing the shielding effectiveness of objects of various shapes under conditions that approximate real-world applications.
4. Near-Field Probe Method: This method employs a near-field probe positioned close to the surface of the object under test to measure the distribution of the electromagnetic field on the object’s surface. The electromagnetic shielding performance of the object is assessed by analyzing the attenuation of the electromagnetic field. With its high-resolution detection capability, this method can rapidly identify localized electromagnetic leakage points in electronic devices.

Considering comprehensively the applicability of the test environment, operational convenience, and measurement accuracy, domestic institutions predominantly adopt the shielded room method and the coaxial transmission line method for electromagnetic shielding testing. Depending on the structural characteristics of the test object and specific testing requirements, the coaxial transmission line method can be further subdivided into various forms [97,98,99,100], including the standard coaxial type, flange coaxial type, parallel-plate coaxial type, and waveguide-to-coaxial transition type. These variations collectively ensure accurate evaluation of electromagnetic shielding performance.

In summary, electromagnetic shielding technology achieves effective attenuation of electromagnetic waves through mechanisms such as reflection, absorption, and multiple reflections, thereby preventing electromagnetic interference. Different electromagnetic shielding testing methods each have their own characteristics and applicable scopes. By selecting the appropriate method based on specific testing requirements and application scenarios, the electromagnetic shielding performance of materials and devices can be accurately evaluated, ensuring the stable operation and electromagnetic compatibility of electronic systems.

## 4. Research Progress on Liquid Metal-Based Electromagnetic Shielding Materials

The thickness of shielding materials plays a critical role in determining electromagnetic interference shielding effectiveness (EMI SE), especially in the context of the miniaturization of electronic devices. Thanks to their unique advantages, liquid metal-based electromagnetic shielding materials have become one of the focal points in current research [101,102]. The following provides a brief review of the research progress on liquid metal-based electromagnetic shielding materials.

### 4.1. Electromagnetic Functional Materials Based on Pure Liquid Metal as a Single Filler

Liquid metals with high electrical conductivity can effectively reflect and absorb electromagnetic waves, thereby achieving excellent electromagnetic shielding performance. This makes them highly promising for a wide range of applications in electromagnetic shielding and flexible electronics. Liao et al. [103] employed liquid metal as the sole conductive filler and cellulose nanofibers as the matrix to fabricate LM/CNF composite shielding films through freeze-drying followed by mechanical pressing. In their experiments, the electromagnetic interference shielding effectiveness (EMI SE) increased from 0.3 dB to 38.5 dB as the LM content rose from 0 wt.% to 80 wt.%. Additionally, the EMI SE of the composite films exhibited a positive correlation with frequency. The LM/CNF films, characterized by a layered architecture that facilitates multilevel electron transport pathways, achieved an electrical conductivity of 96,000 S/m. Over a broad frequency range of 4–18 GHz, the EMI SE reached up to 65 dB. Notably, even after thermal treatment at 120 °C for 8 h, the EMI SE remained stable.

**Figure 6:**
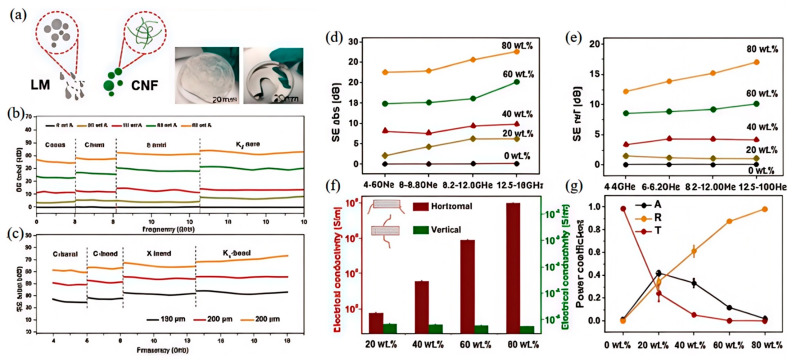
LM/CNF composite films with various LM loadings: (**a**) Raw materials of LM and CNF; (**b**) total electromagnetic interference (EMI) shielding effectiveness of LM/CNF films with an average thickness of 100 μm at different LM loadings; (**c**) total EMI shielding effectiveness of LM/CNF–80 wt.% films with varying thicknesses (from 100 to 300 μm); (**d**) absorption loss and (**e**) reflection loss of 100 m-thick LM/CNF films over a broadband frequency range of 4–18 GHz; (**f**) electrical conductivity of 100 μm–thick LM/CNF films measured in horizontal and vertical directions; (**g**) average absorption loss, reflection loss, and transmission loss of 100 μm–thick LM/CNF films across the 4–18 GHz frequency range. Reprinted/adapted with permission from Ref. [102]. Copyright 2021, Si-Yuan, L.; Xiao-Yun, W.; Xing-Miao, L.; Yan-Jun, W.; Tao, Z.; You-Gen, H.; Peng-Li, Z.; Rong, S.; Ching-Ping, W. More details about “Copyright and Licensing” are available via the following link: https://doi.org/10.1016/j.cej.2021.129962.

However, this study has limitations. From Figure 6f, electrical conductivity shows a significant difference between the horizontal and vertical directions. In practical applications, such a difference may lead to inconsistent electromagnetic shielding effects and electronic transmission performance across different directions. This inconsistency can impact product stability and reliability. Further research is thus needed to optimize performance.

**Figure 7:**
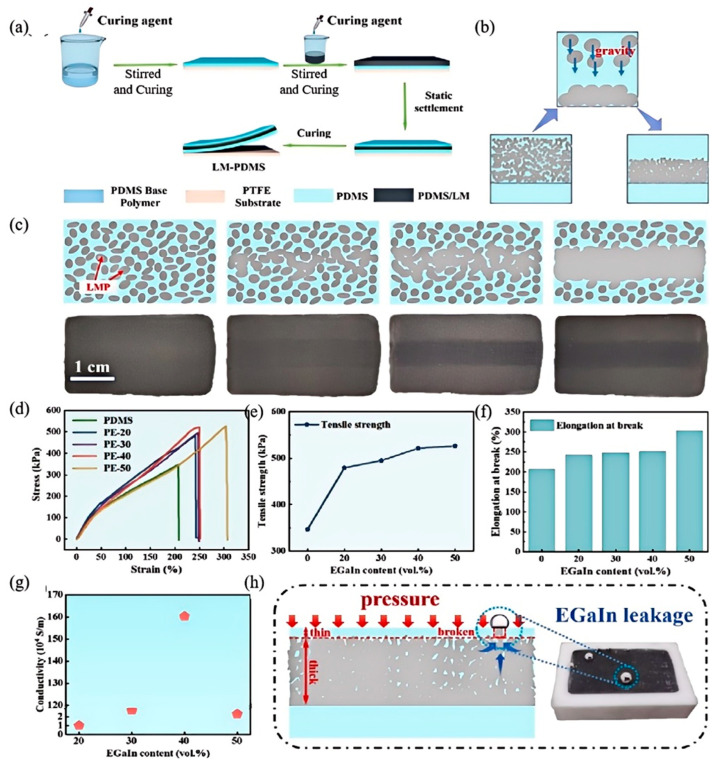
Preparation and characterization of PDMS/EGaIn composite elastomers: (**a**) Schematic of the composite preparation process; (**b**) mechanism of liquid metal (LM) sedimentation and enrichment during curing; (**c**) activation process enabling conductivity in cured composites; (**d**) representative stress-strain curves; (**e**) tensile strength and (**f**) fracture elongation of composites with increasing EGaIn content; (**g**) electrical conductivity versus EGaIn loading; (**h**) schematic illustrating LM leakage mechanisms under mechanical deformation. Reprinted/adapted with permission from Ref. [3]. Copyright 2025, Liu, Z.; Liu, L.; Guan, L.; Zhu, Y.; Lin, M.; Li, Q.; Guo, X.; Wang, Z.; Lian, Y.; Chen, P. More details about “Copyright and Licensing” are available via the following link: https://doi.org/10.1016/j.jmst.2024.12.101.

Liquid metal exhibits excellent fluidity and electrical conductivity. Liu et al. [3] utilized and demonstrated PDMS/EGaIn composite elastomers featuring high flexibility, stable conductivity (<10% resistance variation), and consistent EMI shielding under 100% strain. As shown in Figure 7a–c, fabrication involves shear-controlled deposition of EGaIn droplets in PDMS followed by self-encapsulation. The particle size of EGaIn is tuned via shear velocity/duration, yielding optimal precipitation diameters with 30 min of processing. Mechanical enhancement arises from EGaIn particles suppressing maximum principal strain localization. These particles reinforce the elastomer by enhancing energy dissipation and redirecting crack propagation perpendicularly, thereby mitigating crack- tip stress concentration. This mechanism enables increased fracture elongation (up to 850%) and tensile strength (1.8 MPa) at higher LM volume fractions.

The internally formed defect-free LM network underpins two key functionalities: direction-independent resistance during deformation; and stable EMI shielding (>60 dB) under cyclic strain. Surface circuit inscription is enabled through selective activation. Conductivity scales with EGaIn loading (Figure 7g), reaching 2.4 × 10^4^ S/m at 70 vol%. Leakage prevention is achieved by maintaining droplet sizes below the Rayleigh–Plateau instability threshold (Figure 7h), where differing PDMS/EGaIn surface tensions prevent coalescence. The synergistic integration of mechanical robustness, electrical stability, and manufacturable processing establishes this composite as a versatile platform for stretchable electronics.

However, this design still faces limitations in practical scenarios. As shown in Figure 7h, while controlling EGaIn droplet size below the critical threshold suppresses leakage under standard conditions, extreme pressures or prolonged cyclic loading beyond the design envelope can elevate leakage risks. In real-world applications, complex stress environments accelerate such damage, complicating precise leakage control. As observed in Figure 7g, inconsistent EGaIn particle size and distribution within the PDMS matrix may undermine the reliability of stretchable electronics. Although higher LM volume fractions enhance mechanical properties per current research, long-term stability under complex service conditions is yet to be validated.

**Figure 8:**
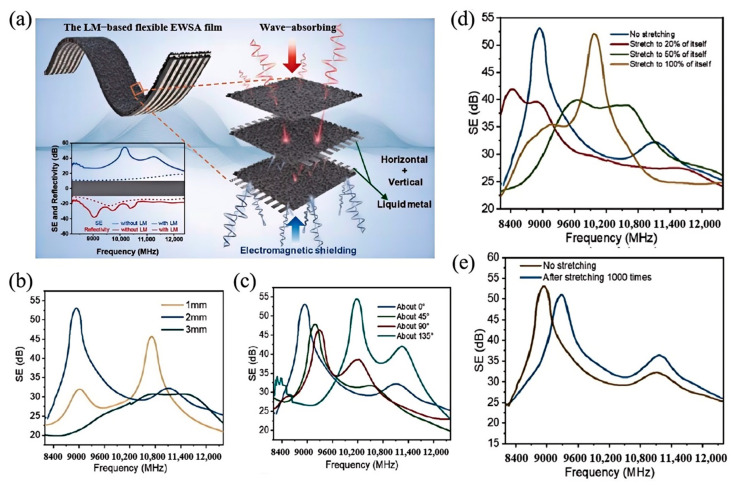
Electromagnetic wave shielding absorber (EWSA) film: (**a**) Schematic illustration of the LM-based EWSA film; (**b**) SE values of films with circuit widths of 1, 2 and 3 mm at a circuit spacing ratio of 1:1; (**c**) SE values of the EWSA film rotated at 0°, 45°, 90° and 135°; (**d**) S-parameters of the EWSA film under 0%, 20%, 50% and 100% tensile strain with a circuit spacing ratio of 1:1 and a width of 2 mm; (**e**) SE values of the EWSA film in arbitrary directions after 1000 stretching cycles. Reprinted/adapted with permission from Ref. [104]. Copyright 2024, Zhang, X.; Deng, Z.; Song, H.; Guo, M.; Li, L. More details about “Copyright and Licensing” are available via the following link: https://doi.org/10.1007/s40843-024-3111-2.

Zhang et al. [104] effectively addressed both internal and external electromagnetic interference (EMI) by printing liquid metal circuits onto Ecoflex@Fe films. As illustrated in Figure 8a, the electromagnetic wave shielding absorber (EWSA) film consists of three layers of Ecoflex@Fe film and two mutually perpendicular liquid metal circuits. During testing, the EWSA film maintained high shielding efficiency and strong wave absorption capability even after undergoing 1000 stretching cycles. Figure 8b presents the measured shielding effectiveness of the EWSA films fabricated with circuit widths of 1 mm, 2 mm and 3 mm. The results indicated that the film with a 2 mm-wide circuit achieved an optimal SE of approximately 53 dB at 9 GHz. Additionally, it was observed that a larger printed area of the liquid metal corresponded to better shielding performance. By altering the structural orientation of the EWSA film, as depicted in Figure 8c, the film was rotated at angles of 0°, 45°, 90° and 135°. When rotated to 0°, over 90% of the electromagnetic waves were reflected. At 45° and 135°, approximately 80% of the waves were reflected. At a 90° rotation, roughly half of the incident radiation at 9.5 GHz was absorbed, revealing that the EWSA film allows significant wave penetration at this orientation. Furthermore, stretching the EWSA film demonstrated effective electromagnetic shielding both during and after the stretching process.

The excellent fluidity and high electrical conductivity of liquid metal have effectively addressed the conventional issues of filler agglomeration and interfacial incompatibility in current studies. However, challenges such as high production costs and the limited reliability of encapsulation materials still hinder its broader application. Overcoming these obstacles will be essential for the future scalable utilization of liquid metal as a single-filler material.

### 4.2. Electromagnetic Functional Materials Combining Liquid Metal with Conductive Fillers

Liquid metals, characterized by ultra-high electrical conductivity, fluidity, and ductility, when combined with conductive fillers, exhibit synergistic effects that surpass the sum of their individual contributions in both performance and functionality. However, the presence of electromagnetic interference (EMI) in liquid metals can easily distort current and voltage waveforms, thereby reducing device stability. Currently, the primary approach to mitigate EMI involves employing electromagnetic shielding technologies. In such systems, conductive fillers serve as a conductive framework for the liquid metal, enhancing the scattering and absorption of electromagnetic waves within the material.

**Figure 9:**
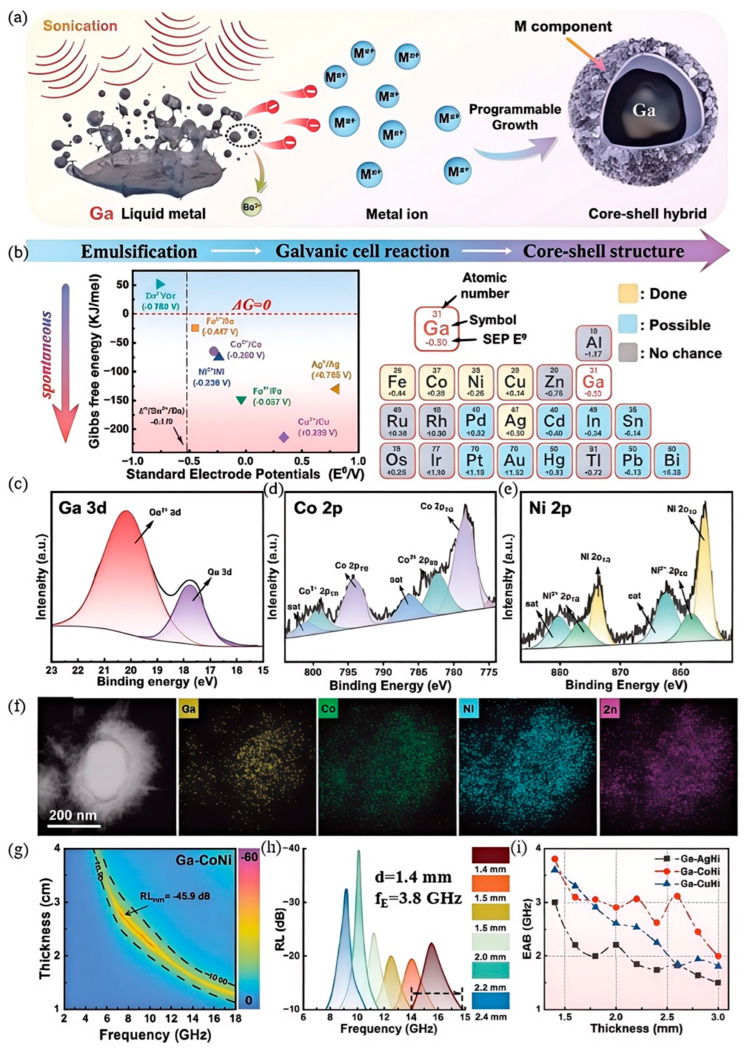
Schematic illustration and characterization of Ga-based liquid metal core–shell nanohybrids: (**a**) Fabrication schematic depicting emulsification via sonication-induced shear within a metal salt solution, followed by galvanic replacement between dispersed Ga droplets and metallic cations; (**b**) calculated Gibbs free energy profiles for redox pairs involving the Ga core and prospective shell metals; (**c**–**e**) high-resolution XPS spectra of the Ga-CoNi nanohybrid: (**c**) Ga 3d, (**d**) Co 2p, (**e**) Ni 2p; (**f**) HAADF-STEM image and corresponding elemental maps (Ga, Co, Ni) for the Ga-CoNi sample; (**g**,**h**) reflection loss characteristics of the Ga-CoNi nanohybrid at varying thicknesses; (**i**) effective absorption bandwidth (EAB) comparison for core–shell Ga-AgNi, Ga-CoNi, and Ga-CuNi nanohybrids across absorber thicknesses of 1.4–3.0 mm. Reprinted/adapted with permission from Ref. [112]. Copyright 2023, Zhao, B.; Du, Y.; Lv, H.; Yan, Z.; Jian, H.; Chen, G.; Wu, Y.; Fan, B.; Zhang, J.; Wu, L. More details about “Copyright and Licensing” are available via the following link: https://doi.org/10.1002/adfm.202302172.

Investigations reveal that pristine gallium-based liquid metal presents an extraordinarily elevated surface tension, registering as high as 718 mN/m [105,106,107]. Under typical ambient conditions, because of their substantial surface tension, liquid metals naturally draw themselves into nearly perfect spheres, posing significant challenges in precise material manipulation and processing. Meanwhile, gallium-based liquid metals exhibit relatively lower surface tension and superior electrical conductivity [14,28,108]. Gallium-based liquid metals demonstrate superior stability in terms of both physical and chemical properties compared to pure gallium. Consequently, recent research has predominantly concentrated on investigating the electromagnetic shielding performance of gallium-based liquid metal materials [33,109,110,111]. Zhao et al. [112] demonstrate a sonication-assisted galvanic replacement strategy (Figure 9a), where probe sonication applies shear forces to bulk Ga-based liquid metal in metal ion solutions, disrupting the native oxide skin. This exposes high-energy surfaces, fragmenting the bulk into spherical droplets. Simultaneously, galvanic replacement occurs as fresh Ga contacts metal ions with higher reduction potentials (e.g., Co^2+^/Co, Ni^2+^/Ni), driving interfacial precipitation to form core–shell structures (liquid metal core/deposited shell). Structural analysis via HAADF-STEM and elemental mapping confirms Ga localization in core regions while bimetallic components (Co/Ni) occupy peripheral shells. XPS survey spectra (referenced in Figure 9f) detect Ga, Ni, Co, Zn, and O in Ga-CoNi, verifying bimetallic shell formation. High-resolution XPS reveals dual metallic/oxidized states of Ga, Co and Ni, indicating minor surface oxidation during synthesis. This approach enables programmable synthesis of diverse nanohybrids (Ga-CoNi, Ga-AgNi, Ga-CuNi, etc.) through tailored ion selection, establishing a versatile platform for tunable core–shell nanostructures. The obtained core–shell nanostructures are used as efficient microwave absorbers to dissipate unwanted electromagnetic wave pollution. The effective absorption bands (90% absorption) of core–shell Ga–Ni and Ga–CoNi nanohybrids are 3.92 and 3.8 GHz at a thickness of 1.4 mm, respectively.

This ultrasound-assisted electrochemical replacement strategy has broad applicability for preparing core–shell nanohybrids. However, it has significant limitations. Precisely controlling ultrasound parameters and metal ion concentrations is a major challenge. From an application view, the narrow absorption bandwidth and high thickness sensitivity limit its use in electromagnetic pollution scenarios. Also, the complex process and high costs hinder its adoption in practical electromagnetic interference shielding. Further optimization is needed.

**Figure 10:**
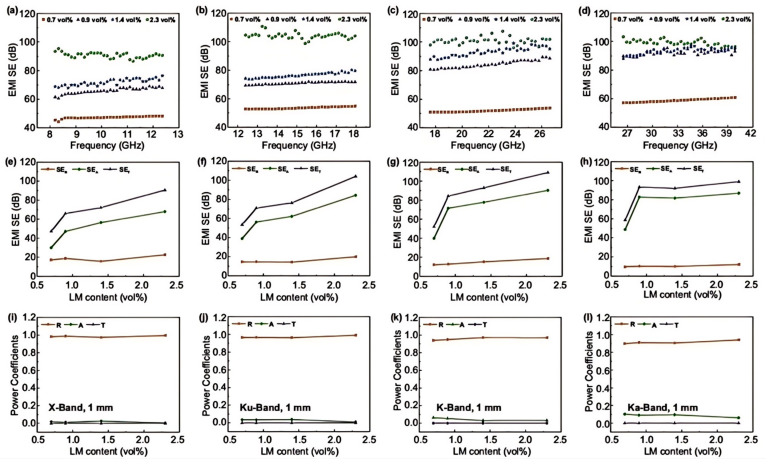
EMI SE performance and shielding mechanisms of EM/LM composites with varying LM contents and a thickness of 1 mm across the 8.2–40 GHz frequency range. (**a**–**d**) EMI SE performance and shielding mechanisms in different frequency bands; (**e**–**h**) comparison of average *SE_R_*, *SE_A_*, and *SE_T_* values, illustrating the EMI shielding mechanisms across various frequency ranges; (**i**–**l**) average power coefficients of reflection, absorption and transmission for EM/LM composites within different frequency ranges. Reprinted/adapted with permission from Ref. [113]. Copyright 2021, Xu, Y.; Lin, Z.; Rajavel, K.; Zhao, T.; Zhu, P.; Hu, Y.; Sun, R.; Wong, C.-P.; Zhang, J.; Wu, L. More details about “Copyright and Licensing” are available via the following link: https://doi.org/10.1007/s40820-021-00766-5.

Xu et al. [113] employed thermally expandable microspheres (EM) in situ to drive the refined and ordered distribution of liquid metal (LM, eutectic indium gallium), achieving a balance between macroscopic stability and microscopic fluidity of the LM conductive network. As illustrated in Figure 10, the EM/LM composite foam, featuring a unique internal gas-filled honeycomb closed-cell structure and a highly conductive LM network, exhibits low density (0.104 g·cm^−1^), high compressive strength (3.43 MPa), excellent compressibility and resilience (with a recovery rate exceeding 90% at 90% compressive strain), and outstanding electrical conductivity (7891 S·m^−1^). Additionally, with an extremely low LM content (2.3 vol%), the EM/LM composite foam achieves an average electromagnetic interference shielding effectiveness (EMI SE) of 98.7 dB across a broad frequency range of 8.2–40 GHz, demonstrating excellent electromagnetic sealing performance.

In this study, the in situ thermal expansion of EM was utilized to precisely control the ordered distribution of eutectic indium gallium (LM), successfully developing an EM/LM composite foam material that integrates lightweight properties, high compressive strength, superior resilience, excellent electrical conductivity and remarkable EMI shielding performance. This advancement not only provides new insights into the design of high-performance composite materials but also opens up promising opportunities for practical applications in related fields.

**Figure 11:**
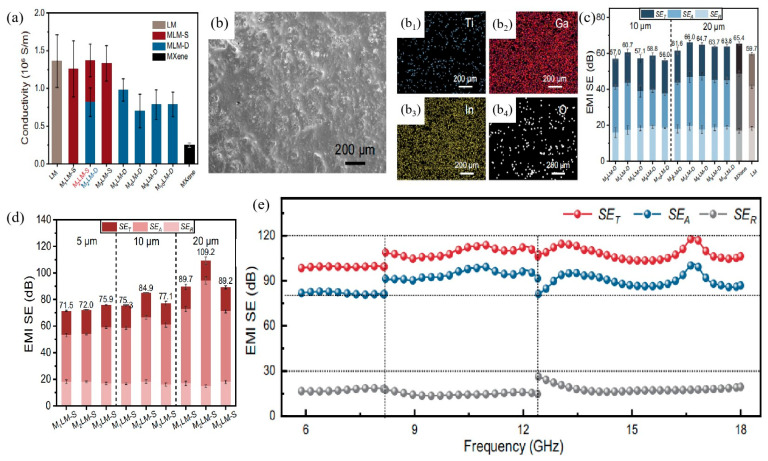
MXene/LM composites prepared by dry mixing and solvent-assisted dispersion (MLM-S): (**a**) Electrical conductivity versus MXene loading for MLM-D and MLM-S coatings; (**b**) top-view SEM micrograph of the MLM-S coating; panels (**b_1_**–**b_4_**) give the corresponding EDS elemental maps; (**c**) MLM-D coatings 10 μm and 20 μm thick; (**d**) MLM-S coatings 5 μm, 10 μm and 20 μm thick; (**e**) broadband (5.88–18 GHz) *SE_T_*, *SE_A_* and *SE_R_* of a 20 μm MLM-S coating. Reprinted/adapted with permission from Ref. [101]. Copyright 2024, Jiang, H.; Yuan, B.; Guo, H.; Pan, F.; Meng, F.; Wu, Y.; Wang, X.; Ruan, L.; Zheng, S.; Yang, Y. More details about “Copyright and Licensing” are available via the following link: https://doi.org/10.1038/s41467-024-50541-4.

Jiang et al. [101] uniformly integrated MXene into EGaIn (75% Ga and 25% In, ≥99.99%), achieving superior internal connectivity compared to conventional dry powder mixing methods (MLM-D). Experimental results revealed that MXene/LM coatings exhibited excellent electromagnetic interference (EMI) shielding performance, reaching approximately 105 dB at a coating thickness of 20 μm, which is about 1.6 times higher than that achieved by traditional dry mixing methods, as illustrated in Figure 11a. Upon incorporation of MXene, the electrical conductivity of LM remained almost unaffected, whereas MLM-D showed a noticeable decrease in conductivity compared to pristine LM. As the MXene loading rose, the EMI shielding effectiveness of MLM-D steadily increased; however, once the MXene fraction surpassed 4 wt.%, the trend levelled off. Beyond this threshold, excessive MXene addition led to particle aggregation during the experiment, resulting in a synergistic effect of less than the sum of individual contributions (1 + 1 < 2). As the coating thickness increased from 10 μm to 20 μm, peak X-band EMI shielding effectiveness climbed from 60.7 dB to 66.0 dB on average, as depicted in Figure 11c. Additionally, Figure 11d indicates that with increasing coating thickness, the absorption loss (*SE_A_*) of MLM-S coatings progressively increased, while reflection loss (*SE_R_*) and total shielding effectiveness (*SE_T_*) remained nearly constant. MXene/LM composite coating materials demonstrate significant advantages in EMI shielding applications. Future research could further optimize MXene content and coating thickness to achieve even higher shielding performance and broader application prospects.

However, the strategy has inherent limitations. As shown in Figure 11a, while MXene/LM maintains favorable conductivity, MLM-D exhibits a notable conductivity decline compared to pure LM. Conductivity variations among samples also show that the mixing process is hard to control stably. For MXene loading, exceeding 4 wt.% causes particle aggregation. This affects shielding performance and increases difficulties in material preparation and uniformity control. In practice, overly thick coatings may limit application scenarios and bring practical risks. Future research can optimize MXene content and coating thickness. This helps achieve higher shielding performance and broader prospects while addressing these limitations.

### 4.3. Electromagnetic Functional Materials Based on the Combination of Liquid Metal and Magnetic Substances

**Figure 12:**
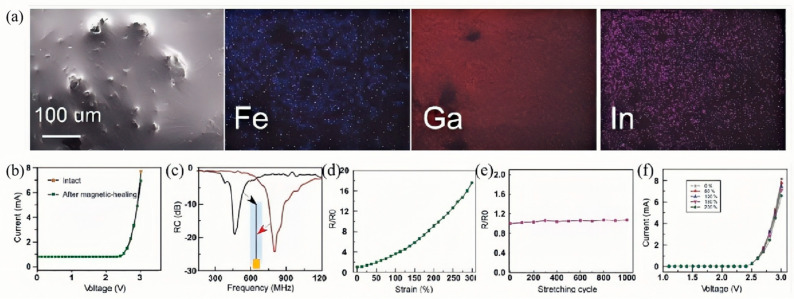
Surface morphology and electrical performance of Fe-EGaIn electronic devices. (**a**) Scanning electron microscopy (SEM) image of Fe-EGaIn and corresponding elemental mappings of Fe, Ga, and In. (**b**) Current–voltage curves of an Fe-EGaIn wire connected to an LED before and after magnetic healing. (**c**) Frequency-dependent reflection coefficient of a reconfigurable antenna measured before and after magnetic healing. (**d**) Resistance variation of a stretchable Fe-EGaIn wire under different strains (up to 300%). (**e**) Resistance variation of a stretchable Fe-EGaIn wire during repeated stretching cycles (up to 1000 cycles). (**f**) I–V curves of an Fe-EGaIn wire connected to an LED under various strains (0–200%). Reprinted/adapted with permission from Ref. [114]. Copyright 2019, Rui, G.; Xuyang, S.; Bo, Y.; Hongzhang, W.; Jing, L. More details about “Copyright and Licensing” are available via the following link: https://doi.org/10.1002/advs.201901478.

Electromagnetic functional composites formed by dispersing liquid metal together with magnetic agents, such as iron powder or Fe_3_O_4,_ realize multidimensional performance breakthroughs and intelligent responsiveness. The leap in capability arises from the complementary strengths of the metallic fluid’s pronounced electrical conductivity and inherent fluidity, coupled with the immediate magnetic reactivity of the added magnetic constituents. Guo et al. [114] introduced iron powder at a predetermined mass fraction into a gallium–indium alloy, thereby creating a magnetic liquid metal paste. Experimental observations showed that the incorporation of solid iron particles noticeably restrained the alloy’s flow, which in turn elevated its formability and printability for practical deployment. Under an external magnetic field, the motion of the embedded iron particles was governed with remarkable accuracy. Figure 12 illustrates that the parent liquid metal, governed by its inherently high surface tension and non-magnetic nature, remains unresponsive to magnetic influences; consequently, broken conductive traces cannot be actuated or autonomously restored. In contrast, after blending with iron powder, the composite maintained stable electrical resistance even after undergoing 1000 cycles of 100% strain testing. Furthermore, by integrating an LED lamp with an Ecoflex substrate and connecting it with Fe-EGaIn wires, the system demonstrated remarkable stability under varying strains ranging from 0% to 200%, without compromising circuit functionality.

However, inherent limitations persist. As shown in Figure 12a, uneven iron powder distribution within the alloy can induce inconsistencies in the composite’s mechanical and electrical properties. This affects the reliability of the paste in large-scale or high-precision applications. The composite showed stable performance in 1000 cycles of 100% strain testing. But prolonged exposure to more extreme strains or high-frequency cyclic strain may still induce fatigue damage. Notably, its performance in complex environments (e.g., high temperature, humidity or corrosion) remains unclear. This may limit future practical applications and require further research.

**Figure 13:**
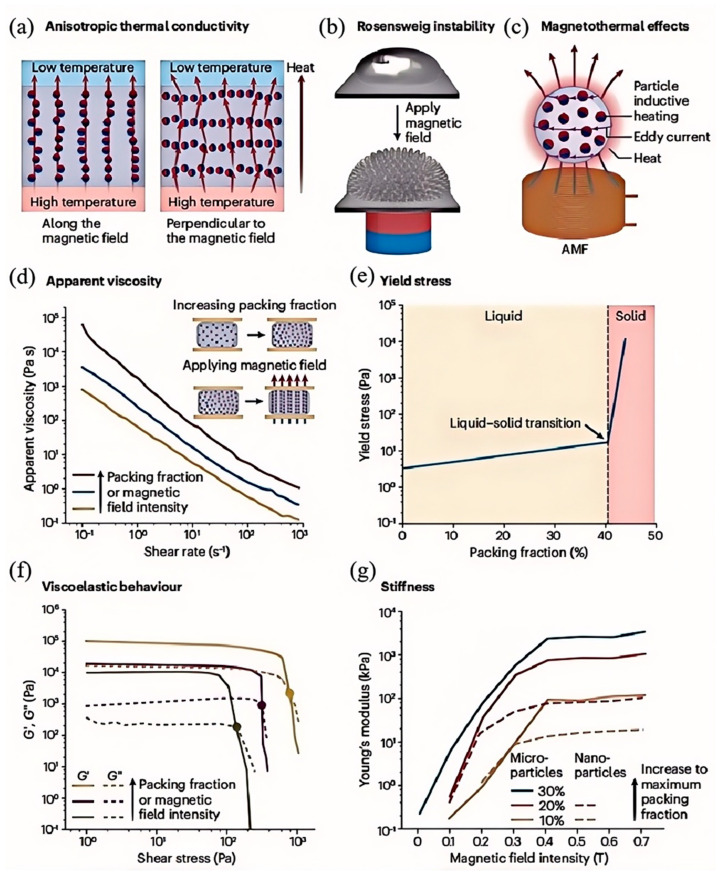
Liquid metal-based magnetic fluids (LMMFs): (**a**) Anisotropic thermal conductivity of LMMFs under a magnetic field, exhibiting higher thermal conductivity along the magnetic field direction; (**b**) Rosensweig instability phenomenon, where a vertically applied uniform magnetic field induces sharp peak-like deformations in LMMFs; (**c**) magnetothermal effect of LMMFs, resulting from eddy currents in the liquid metal and inductive heating of magnetic particles under alternating magnetic fields; (**d**) apparent viscosity of LMMFs increases with higher magnetic particle loading and applied magnetic field strength; (**e**) yield stress of LMMFs as a function of magnetic particle loading, with a significant increase indicating a transition from liquid-like to solid-like behavior; (**f**) viscoelastic behavior of LMMFs varying with particle loading and magnetic field intensity; (**g**) stiffness of LMMFs positively correlates with particle loading, particle size, and magnetic field strength, with particle diameter directly influencing the maximum achievable loading fraction. Reprinted/adapted with permission from Ref. [115]. Copyright 2024, Wentao, X.; Yongyu, L.; Hongzhang, W.; Xuyang, S.; Sen, C.; Zhizhu, H.; Jing, L. More details about “Copyright and Licensing” are available via the following link: https://doi.org/10.1038/s41578-024-00679-w.

Xiang et al. [115] replaced conventional carrier fluids such as oil or water with liquid metal to suspend magnetic particles like Fe_3_O_4_, resulting in a magnetically functional liquid metal dispersion whose electrical conductivity, colloidal stability, and resilience to elevated temperatures were all substantially improved. Laboratory evaluations recorded an electrical conduction value surpassing 10^4^ S m^−1^, together with a magnetic response time roughly triple that of standard ferrofluids. The continuous metallic phase inherently provides the dispersion with outstanding thermal and electrical transport characteristics. When exposed to a magnetic field, the magnetized particles attract each other and align along the field direction to form chain-like structures, thereby increasing the fluid’s viscosity. Additionally, it was observed that the apparent viscosity of the liquid metal magnetic fluid exhibited low temperature sensitivity, as the motion of magnetic particles—especially larger ones—was largely unaffected by temperature variations, as shown in Figure 13e. With increasing particle loading, the yield stress of the liquid metal magnetic fluid initially increased gradually and then rose sharply, indicating a transition from a liquid-like to a solid-like state. Further experiments revealed that upon application of an external magnetic field, the Young’s modulus (a measure of stiffness) increased by approximately fourfold; once the magnetic field was removed, the Young’s modulus returned to its original value, as depicted in Figure 13e.

However, limitations are evident from structural behavior. Magnetic-field-induced chain-like structures enhance viscosity yet cause uneven stress distribution within the dispersion. Over time, especially with alternating magnetic fields, particles may aggregate or separate locally, reducing long-term performance stability. The sharp increase in yield stress during liquid-to-solid transition hinders precise rheological control in practice. Magnetically induced, Young’s modulus’ changes offer tunable stiffness, but rely on a continuous external magnetic field.

**Figure 14:**
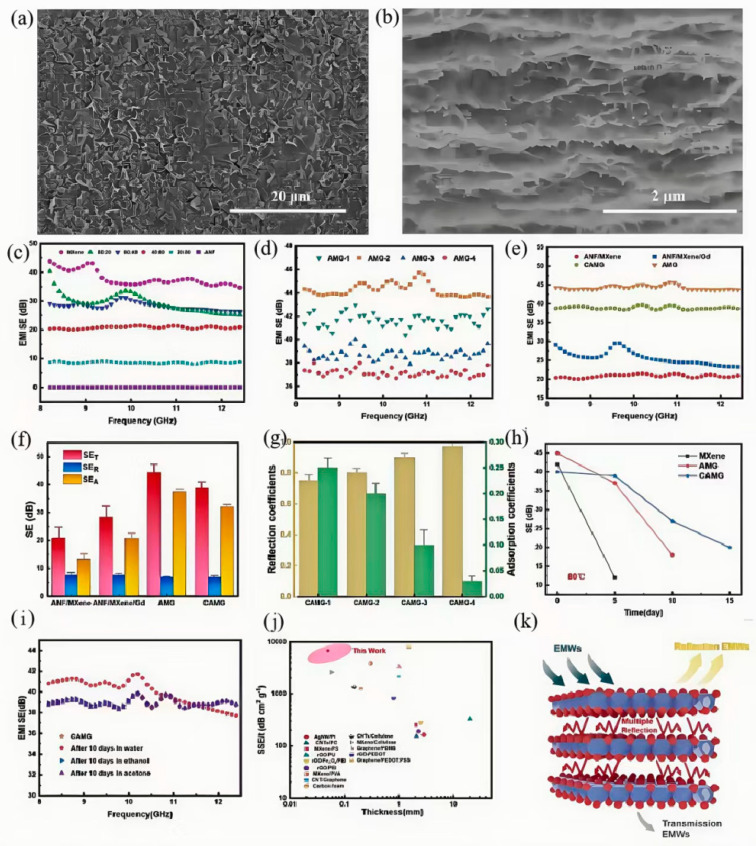
Aramid nanofiber-based nanocomposite films: (**a**) Scanning electron microscopy (SEM) image of the CAMG film surface; (**b**) cross-sectional SEM image of the CAMG film; (**c**) electromagnetic interference (EMI) shielding effectiveness of the ANF-MXene composite film in the X-band (8.2–12.4 GHz); (**d**) EMI shielding effectiveness of the AMG composite film; (**e**) comparison of EMI shielding effectiveness among different composite films; (**f**) total shielding effectiveness (*SE_T_*), reflection loss (*SE_R_*), and absorption loss (*SE_A_*) values of various composite films at 12.4 GHz; (**g**) reflection–absorption coefficients of the CAMG composite film in the X-band; (**h**) time-dependent shielding effectiveness of MXene, AMG, and CAMG films stored at 80 °C; (**i**) EMI shielding effectiveness of the CAMG composite film after immersion in different solvents; (**j**) comparison of thickness and specific shielding effectiveness (SSE/t) among various EMI shielding materials; (**k**) schematic illustration of the shielding mechanism of the CAMG composite film. Reprinted/adapted with permission from Ref. [116]. Copyright 2024, Shen, M.; Xu, X.; Qi, J.; Li, X.; Xue, B.; Zhu, M.; Zhang, Z.; Zheng, X.; Li, B.; Shang, Z. More details about “Copyright and Licensing” are available via the following link: https://doi.org/10.1016/j.compositesa.2024.108178.

Shen et al. [116] developed a nanocomposite film composed of aramid nanofibers (ANF), MXene-bridged gallium droplets, and Gd_2_O_3_ as a magnetic loss agent, fabricated via a chemical cross-linking strategy (denoted as CAMG), as shown in Figure 14. The resulting CAMG shielding film features multiple heterogeneous interfaces and a synergistic magnetic-dielectric system, which significantly enhances absorption-dominated shielding efficiency, achieving a remarkable value of 6788 dB·cm^−1^·g^−1^. Although the AMG shielding material (without cross-linking) exhibited improved stability compared to pure MXene films, its electromagnetic interference (EMI) shielding effectiveness still declined noticeably over time. In contrast, the hydrophobic CAMG film, reinforced by chemical cross-linking, maintained structural integrity even after storage at 80 °C for 15 days, retaining a shielding effectiveness of approximately 20 dB. Moreover, the cross-linked CAMG film demonstrated excellent stability in various solvents, including water, ethanol, and acetone.

However, limitations remain in long-term performance and practical application. CAMG shows better stability than AMG. But the long-term effects of prolonged exposure to extreme environments (e.g., high temps, harsh chemicals) on its structure and shielding efficiency are under-studied. The fabrication process is complex and costly. This limits large-scale production and widespread use. Specific components also hinder CAMG’s commercialization. Thus, further research is needed.

### 4.4. Effects of Different Liquid Metal Forming Structures on Electromagnetic Waves

**Figure 15:**
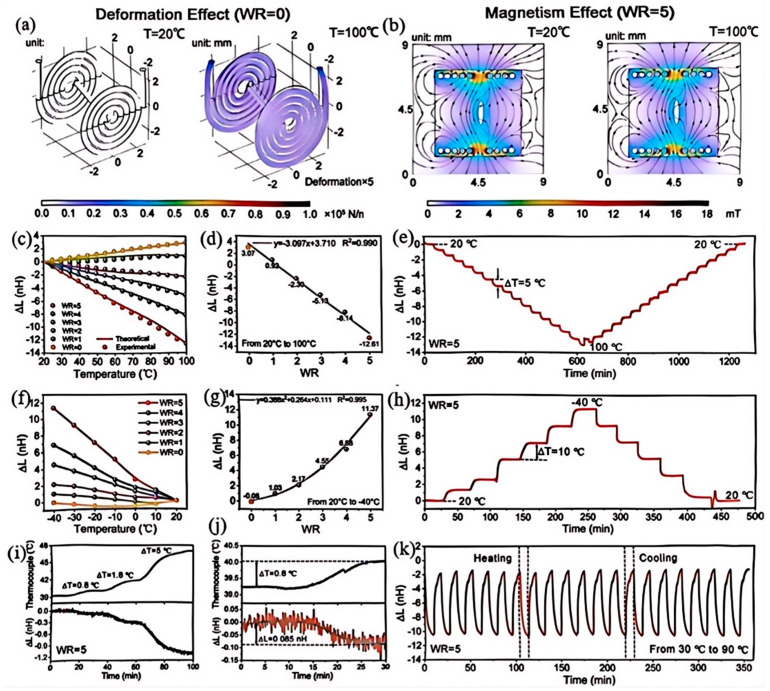
Temperature–response characteristics of the antagonistic liquid metal architecture (ALMA): (**a**) ALMA with WR = 0 exhibits the largest deformation effect; (**b**) ALMA with WR = 5 shows a stronger magnetic response; (**c**) in the high-temperature range (20–100 °C), ALMA signals with different WR values display excellent linearity; (**d**) under high-temperature conditions, variations in WR lead to linear changes in ALMA signals; (**e**) ALMA with WR = 5 undergoes heating-cooling cycles between 20 and 100 °C, with a 30-min hold at every 5 °C interval; (**f**) ALMA also demonstrates temperature-sensing capability in the low-temperature range (20 to −40 °C); (**g**) within the low-temperature range, ALMA with WR = 5 exhibits the largest inductance variation with temperature; (**h**) ALMA with WR = 5 is tested during cooling and reheating between 20 and −40 °C, with a 30-min hold at every 10 °C interval; (**i**) ALMA (WR = 5) shows extremely high sensitivity to minute temperature changes; (**j**) when the thermocouple reading increases by 0.8 °C, ALMA detects an inductance change of 0.085; (**k**) during 30–90 °C thermal cycling tests, ALMA (WR = 5) demonstrates outstanding cyclic stability. Reprinted/adapted with permission from Ref. [117]. Copyright 2025, Li, N.; Zhan, F.; Su, J.; Li, Y.Q.; Chen, X.Q.; Guo, M.H.; Wang, L.; Liu, J. More details about “Copyright and Licensing” are available via the following link: https://doi.org/10.1002/adfm.202507514.

Owing to their unique characteristics, liquid metals have attracted considerable attention in electromagnetic (EM) shielding applications. Different structural configurations can effectively modulate the propagation pathways of EM waves, including reflection, absorption, and scattering, thereby significantly influencing the shielding effectiveness, operational bandwidth, and functional integration capabilities of the resulting materials. Liao et al. [102] employed freeze-drying combined with mechanical pressing to construct a layered architecture. During the process, liquid metal droplets were compressed and ruptured, subsequently forming a continuous conductive network. The presence of this layered configuration effectively extended the propagation path of electromagnetic waves, significantly enhancing energy dissipation and ensuring stable material performance under elevated temperatures. Xu et al. [113] employed thermally expandable microspheres to drive liquid metal into forming a honeycomb-like closed-cell structure under confined expansion conditions, thereby constructing a three-dimensional interconnected conductive network. The presence of closed pores effectively induces multiple internal reflections of electromagnetic waves, enhancing both the conductive losses of the liquid metal and the dielectric losses of the pore walls, which significantly improves the electromagnetic shielding effectiveness of the porous foam-structured liquid metal material. More notably, the multi-cellular foam material exhibits exceptional compressive resilience, greatly expanding its practical applicability. Li et al. [117] developed an antagonistic liquid metal intelligent architecture through biomimetic surface design, leveraging the competing effects between deformation and magnetism, as shown in Figure 15. Their experiments revealed that as temperature rises, thermal expansion of the material matrix alters the geometry of embedded liquid metal coils, resulting in a positive inductive response. Simultaneously, the magnetic permeability of the PDMS@Fe matrix decreases with increasing temperature, leading to a reduction in magnetic field strength. By employing liquid metal crystallization templates to in situ grow ZnO micro/nano structure arrays, the authors successfully constructed a biomimetic superhydrophobic surface for the antagonistic liquid metal architecture (ALMA). Embedding liquid metal coils into the Fe-PDMS matrix and utilizing the antagonistic interplay between thermal expansion and magnetic permeability changes enabled signal self-decoupling; as temperature increases, matrix expansion elevates while permeability declines, thereby achieving autonomous signal modulation. However, inherent limitations emerge from experimental data. The inductive response (ΔL) shows complex dependencies on temperature, WR (a structural parameter), and time. In practical applications with variable environments, precisely controlling ALMA performance is challenging. Repeated thermal expansion and contraction may damage the matrix microstructurally. Over time, this alters its magnetic and mechanical properties. Thus, it reduces the reliability of signal self-decoupling. Further research is needed. It should optimize ALMA’s material stability, fabrication process, and environmental adaptability. This helps achieve more robust and widespread applications.

### 4.5. Research on Electromagnetic Shielding Performance of Liquid Metal-Based Multifunctional Materials

**Figure 16:**
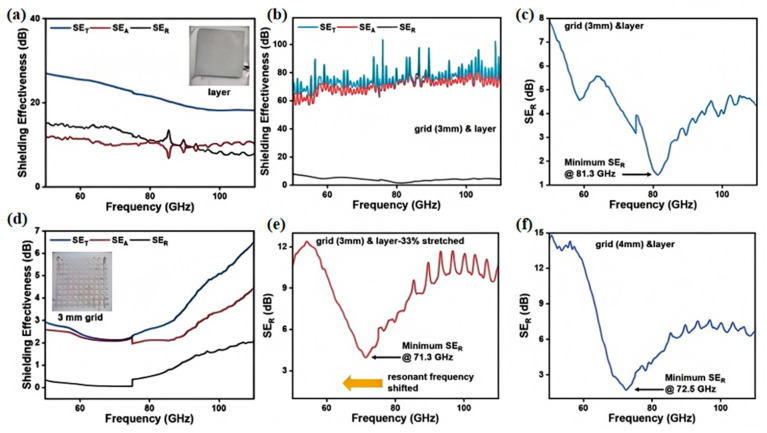
(**a**) EMI SE of the continuous LM layer, 50–110 GHz; (**b**) EMI SE of the LM mesh (3 mm grid), 50–110 GHz; (**c**) EMI SE and (**d**) reflection loss (*SE_R_*) of a 410 µm LMGD with 3 mm grid, 50–110 GHz; (**e**) *SE_R_* of the same 410 µm LMGD under 33% strain, 3 mm grid, 50–110 GHz; (**f**) EMI SE of a 410 µm LMGD with 4 mm grid, 50–110 GHz. Reprinted/adapted with permission from Ref. [118]. Copyright 2024, Wei, Y.; Bhuyan, P.; Kwon, S.J.; Kim, S.; Bae, Y.; Singh, M.; Tran, D.T.; Ha, M.; Jeong, K.-U.; Ma, X.; et al. More details about “Copyright and Licensing” are available via the following link: https://doi.org/10.1007/s40820-024-01457-7.

At present, liquid metal-based shielding materials have evolved from single-functionality toward multifunctionality, with lightweight and stretchable properties gradually becoming key development objectives, particularly for applications under various environmental conditions. Wei et al. [118] fabricated a soft and stretchable EMI shielding thin-film device with excellent shielding performance. The device consists of a liquid metal (LM) layer and a mesh-patterned layer, separated by a thin elastic membrane, as shown in Figure 16. Experimental results demonstrate that the device achieves outstanding electromagnetic shielding effectiveness, reaching up to 75 dB, while maintaining extremely low reflectivity. At the resonant frequency, the shielding effectiveness due to reflection (*SE_R_*) is only 1.5 dB. When evaluating the shielding performance of the two-layer structure, it was observed that the LM thin film exhibited relatively high electromagnetic interference (EMI) levels when its shielding effectiveness exceeded 20 dB, whereas the LM mesh pattern generated almost no EMI at the resonant frequency. With a mesh spacing of 3 mm, the device demonstrated exceptional shielding capability across a frequency range of 50 GHz to 110 GHz, achieving a shielding effectiveness of 75 dB. Notably, at 81.3 GHz, the minimum *SE_R_* was merely 1.4 dB.

Additionally, experiments demonstrated that applying tensile strain parallel to the electrode direction enables modulation of the mesh spacing within the LM patterned layer. When the device was stretched to 33% strain, its resonant frequency shifted from 81.3 GHz to 71.3 GHz. To verify this shift, a second device with an initial 4 mm mesh spacing was tested and showed a peak at 72.5 GHz. This reproducible, stretch-induced tuning demonstrates its potential as a stretchable electromagnetic shield for broad applications.

**Figure 17:**
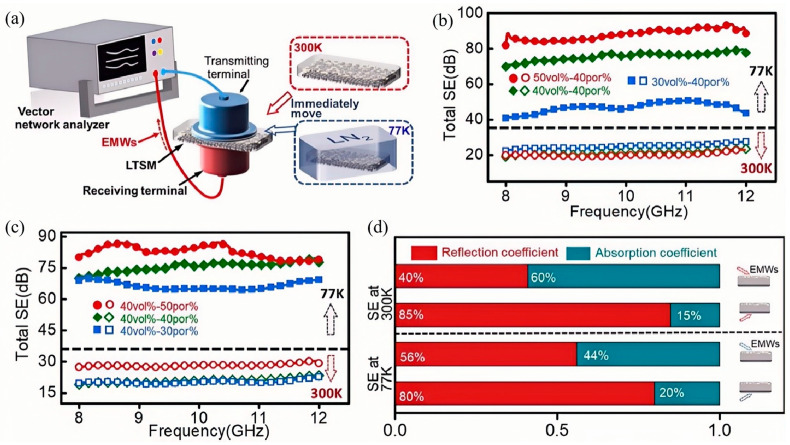
(**a**) EMI SE of the LM layer for electromagnetic waves in the 50–110 GHz band; (**b**) EMI SE of the LM mesh pattern with a 3 mm grid spacing in the 50–110 GHz band; (**c**) EMI SE and (**d**) reflection loss (*SE_R_*) of a 410 µm-thick LMGD with a 3 mm grid spacing in the 50–110 GHz band. Reprinted/adapted with permission from Ref. [119]. Copyright 2020, Zhang, M.; Zhang, P.; Zhang, C.; Wang, Y.; Chang, H.; Rao, W. More details about “Copyright and Licensing” are available via the following link: https://doi.org/10.1016/j.apmt.2020.100612.

Zhang et al. [119] successfully developed a novel porous-structured material based on gallium-based liquid metal (LM) and polydimethylsiloxane (PDMS) through a combination of solution blending and thermal treatment. The design concept of this material stems from the growing demand for high-performance electromagnetic interference (EMI) shielding materials, particularly for applications under extreme environmental conditions. As shown in Figure 17, when the mass fraction of gallium-based liquid metal reaches 40%, this porous composite material exhibits outstanding EMI shielding performance even under cryogenic conditions (77 K). Specifically, within the X-band frequency range, the material achieved an outstanding EMI shielding effectiveness (SE) of up to 75 dB, significantly surpassing the requirements of military standards. This result highlights the material’s capability to maintain highly efficient EMI shielding under extreme low temperature conditions, which is of great significance for applications in military communications and electronic device protection. Further investigations demonstrated the tunability of the material’s performance. As the content of gallium-based LM increased, the EMI shielding performance of the composite showed a clear upward trend. This enhancement can be attributed to the improved distribution and interfacial interactions of LM within the porous structure. Additionally, due to the inherent deposition characteristics of gallium-based LM, adjusting the incident direction of electromagnetic waves effectively modulated the reflection loss of the material. Such tunability offers a new perspective for designing EMI shielding mechanisms, enabling the material to be optimized according to diverse application scenarios and specific requirements.

Moreover, the fabrication process has scalability challenges. Solution blending and thermal treatment are difficult to scale for large-scale production. Additionally, gallium-based LM shows long-term instability. It is prone to leakage or agglomeration, which reduces shielding effectiveness. These limitations highlight the need for further research. It should focus on improving the material’s stability, environmental adaptability, and production feasibility.

The aforementioned studies demonstrate that rational structural design and regulation enable the development of diverse high-performance liquid metal-based electromagnetic interference (EMI) shielding materials. These materials not only deliver outstanding shielding effectiveness across broad frequency ranges but also integrate multiple functionalities, including low density, high stretchability, excellent electrical conductivity, and superior thermal conductivity. Such investigations offer novel insights and methodologies for designing advanced EMI shielding materials, particularly for applications under extreme conditions, thereby highlighting their significant potential and promising prospects.

A brief summary of the research progress on liquid metal-based electromagnetic shielding materials is presented in Table 2.

## 5. Prospect

As an emerging high-performance material, liquid metal-based electromagnetic shielding (EMI shielding) materials have achieved remarkable progress in recent years. This progress covers both theoretical research and application development. However, compared with the international advanced level, domestic research in this field still lags behind to some extent. At present, the development of liquid metal-based electromagnetic shielding materials is restricted by four key challenges that need to be solved urgently: First, their shielding effectiveness is insufficient, especially in complex electromagnetic environments, which makes it difficult to meet the protection requirements of high-precision electronic devices. Second, the material stability is poor, and performance degradation will occur when exposed to long-term temperature and humidity fluctuations. Third, the processability is not ideal, and existing processes cannot realize the precise fabrication of products with complex shapes. Fourth, there are great challenges in cost control, as the costs of core raw materials and fabrication equipment remain at a high level, which limits the large-scale application of such materials. To overcome the existing bottlenecks, future research on liquid metal-based electromagnetic shielding materials can focus on the following four directions:

(1) Develop high-performance liquid metal-based materials.

Existing research data further confirms the urgency of addressing the shielding effectiveness issue. There is still significant room for improvement in this aspect. Taking gallium-based liquid metals as an example, their shielding effectiveness in the 1–10 GHz frequency band usually ranges from 40 to 60 dB, yet some aerospace equipment demands a shielding effectiveness of over 80 dB. Therefore, to overcome this gap and the aforementioned bottlenecks, future research on liquid metal-based electromagnetic shielding materials can focus on the following directions: Developing materials with higher shielding effectiveness, broader applicable frequency bands, lower density, and thinner thickness through approaches like component optimization and microstructural control, while also exploring solutions for stability enhancement, processability improvement, and cost reduction.

(2) Develop multifunctional integrated liquid metal-based materials.

Meanwhile, the evolution of modern electronic devices toward miniaturization and higher power output has raised expanded performance requirements for materials—beyond single-function electromagnetic shielding, a multi-dimensional synergy covering “shielding-heat dissipation-mechanical support-sensing” is now needed. Current single-function liquid metal electromagnetic shielding materials can no longer satisfy these demands. Compositing liquid metal with other functional materials (such as high-thermal-conductivity ceramics, flexible polymers, and sensing units) to develop composite materials integrating electromagnetic shielding, thermal management, mechanical strength, and self-sensing functions.

(3) Explore alternative liquid metal systems and their EMI shielding potential.

Current research on liquid metal-based electromagnetic shielding media has primarily focused on gallium-rich alloys and their derivatives (such as gallium–indium alloys and gallium–tin alloys). While these alloys offer advantages such as low melting points and high conductivity, they also present challenges such as high costs and unclear toxicity in certain systems. Future research should prioritize expanding the scope to explore the electromagnetic shielding potential of novel systems such as bismuth-based alloys, sodium–potassium alloys, and low-toxicity alloys. Additionally, easily meltable metal mixtures that can liquefy at moderate temperatures (e.g., 50–100 °C) warrant focused research. These materials offer unique process advantages in temperature-sensitive applications (e.g., medical electronics, food industry equipment) for electromagnetic shielding, as they can avoid damage to equipment caused by high-temperature processing.

(4) Optimize liquid metal composite structures and design novel systems.

On the one hand, it is necessary to explore composite structures combining liquid metals with nanoscale reinforcing materials (such as carbon nanotubes, graphene, and metal nanoparticles). By regulating interfacial bonding strength and optimizing dispersion, the mechanical properties and shielding performance of the material can be enhanced. For example, uniformly dispersing graphene in liquid metal can leverage graphene’s high mechanical strength and high specific surface area to simultaneously improve the material’s tensile strength and electromagnetic loss efficiency. On the other hand, research should be conducted on the design of novel liquid metal systems, such as constructing “liquid metal-aerogel” porous structures or “liquid metal-phase change material” smart responsive structures, to endow the material with unprecedented properties (such as lightweight, adaptive shielding, and self-healing capabilities), thereby opening up new application directions for electromagnetic shielding.

In summary, research on liquid metal-based electromagnetic shielding materials has broad prospects, but its development still faces many challenges. In the future, breakthroughs in four major areas—high-performance material research and development, multifunctional integrated design, exploration of new systems, and optimization of composite structures—are expected to promote the industrialization of such materials, providing key technological support for electromagnetic protection in fields such as electronics, aerospace, and medicine.

## Figures and Tables

**Table 1:** Method for preparing liquid metal-based materials.

Preparation Method	Advantages	Disadvantages	Scalability Potential
Direct Molding Method [58,59,60,61,62,63,64]	Elementary process with simple operation and low equipment cost; Leverages low melting point and high fluidity of liquid metals for easy shaping; High production efficiency via rapid raw material conversion.	Limited to simple geometries (cannot fabricate complex structures); Mold-dependent (additional mold design/manufacturing cost); Internal stress-imbalance risk during natural cooling solidification.	Well-suited for industrial mass production via continuous molding and automated cooling systems; ideal for standardized components.
Composite Preparation Method [65,66,67,68,69,70]	Integrates liquid metal advantages (high conductivity/fluidity) with other materials to enable new/enhanced properties; Versatile for liquid metal–metal/ceramic/polymer composites; Achieves comprehensive properties unavailable in single materials.	Complex process (requires pretreatment, mixing–dispersion, shaping–curing); Interface compatibility issues between components (affects stability); Difficulty in controlling mixing uniformity (causes performance fluctuations).	Mature for lab-scale production; mass production requires breakthroughs in automated mixing equipment and interface optimization; gradually applied in functional materials.
3D Printing Technology [71,72,73,74]	Enables precise fabrication of complex structures; Supports personalized customization with flexible design adjustments; Layer-by-layer deposition enhances material performance via structural control.	High equipment cost and maintenance difficulty; Slow printing speed (low throughput, unsuitable for mass production); Strict requirements on fluidity/stability of liquid metal-material mixtures.	Superior for precision components and personalized products; Mass production needs high-speed printing and cost reduction; Currently for small-batch customization.
Mechanical Stirring [75]	Low maintenance cost; Realizes liquid metal micronization (microdroplets/lamellae) and homogenization.	Impurity introduction risk; Wide particle size distribution.	Suitable for medium-scale powder/microdroplet production; Needs impurity control for scaling.
Microfluidic Control [76]	Micron-level precision; Uniform product size/composition (ideal for micro/nano-composites).	Chip-dependent (high cost); Ultra-low throughput (lab-scale only).	Lab R&D focus; Hard to scale; For micro/nano-device small-batch production.
Vapor Deposition [77]	High-purity films/coatings with good compactness.	Slow deposition rate; Complex equipment (limited to films, not bulk materials).	Small-scale film production; Needs deposition rate improvement for scaling.
Ultrasonic Electroplating Replacement [78]	Metallurgical bonding between substrate and liquid metal; Uniform interface composition.	Narrow process window (strict temperature/immersion time control); Limited substrate applicability.	Suitable for specific substrate coatings; Needs process stability optimization for scaling.

**Table 2:** Research Progress on Liquid Metal-based Electromagnetic Shielding Materials.

	Researchers	Material Systems	Characteristic Advantages	Ref
LM Single filler	Liao et al.	LM/CNF composite shielding film	stable electromagnetic shielding effectiveness of 65 dB	[102]
	Liu et al.	PDMS/EGaIn composite elastomer	high flexibility, stable conductivity (<10% resistance variation), and consistent EMI shielding under 100% strain	[3]
	Zhang et al.	EWSA film	As the effective printing area increases, the shielding performance improves, reaching 53 dB	[104]
LM/ conductive fillers	Zhao et al.	Ga-based liquid metal	core–shell Ga–Ni and Ga–CoNi nanohybrids are 3.92 and 3.8 GHz at a thickness of 1.4 mm, respectively.	[112]
	Xu et al.	EM/LM foam	ultralight and highly resilient, with a shielding effectiveness of 98.7 dB	[113]
	Jiang et al.	MXene/LM	ultra-thin and 3D-printable, with a shielding effectiveness of 105 dB	[101]
LM/ magnetic substances	Guo et al.	Fe-EGaIn magnetic circuit material	magnetic-field-controlled repair, easy-to-shape printing	[114]
	Xiang et al.	LMMFs magnetically controlled material	magnetic fluid: conductivity > 10^4^ S/m, Young’s modulus increased by ~4 times	[115]
	Shen et al.	ANF/MXene@Ga/Gd_2_O_3_ membrane	enables rapid heat dissipation, with a shielding efficiency per unit volume reaching 6788 dB·cm^−1^·g^−1^	[116]
LM different forming structures	Liao et al.	Layered hierarchical structure; LM/CNF composite film	broadband coverage, high-temperature stability, shielding effectiveness of 65 dB (4–18 GHz)	[102]
	Xu et al.	Porous foam structure; EM/LM composite foam	ultrahigh compressive resilience, shielding effectiveness of 98.7 dB (8.2–40 GHz)	[113]
	Li et al.	Dynamic tuning structure; ALMA antagonistic coil	increasing temperature leads to decreased magnetic permeability and dynamic narrowband	[117]
LM-based multifunctional materials	Wei et al.	LM/mesh pattern layer	wide frequency range, lightweight and stretchable, excellent electromagnetic shielding effectiveness	[118]
	Zhang et al.	LM/PDMS novel porous material	high electromagnetic shielding effectiveness under extreme conditions, with tunability	[119]
